# Phase I Trial Evaluating the Safety and Immunogenicity of Candidate TB Vaccine MVA85A, Delivered by Aerosol to Healthy *M.tb*-Infected Adults

**DOI:** 10.3390/vaccines9040396

**Published:** 2021-04-16

**Authors:** Michael Riste, Julia L. Marshall, Iman Satti, Stephanie A. Harris, Morven Wilkie, Raquel Lopez Ramon, Danny Wright, Rachel E. Wittenberg, Samantha Vermaak, Rebecca Powell Doherty, Alison Lawrie, Christopher P. Conlon, Catherine Cosgrove, Fergus Gleeson, Marc Lipman, Paul Moss, Felicity Perrin, Martin Dedicoat, Henry Bettinson, Helen McShane

**Affiliations:** 1 The Jenner Institute, University of Oxford, Oxford OX3 7DQ, UK; michael.riste@nhs.net (M.R.); julia.marshall@ndm.ox.ac.uk (J.L.M.); Iman.Satti@ndm.ox.ac.uk (I.S.); stephanie.harris@ndm.ox.ac.uk (S.A.H.); morvenwilkie@hotmail.co.uk (M.W.); raquel.lopezramon@ndm.ox.ac.uk (R.L.R.); danny.wright@ndm.ox.ac.uk (D.W.); rachel_wittenberg@hms.harvard.edu (R.E.W.); Samantha.Vermaak@NDM.ox.ac.uk (S.V.); rebecca.powelldoherty@ndm.ox.ac.uk (R.P.D.); alison.lawrie@ndm.ox.ac.uk (A.L.); 2 Department of Infection and Tropical Medicine, Heartlands Hospital, Birmingham B9 5SS, UK; martin.dedicoat@heartofengland.nhs.uk; 3 Nuffield Department of Medicine, University of Oxford, Oxford OX3 7DQ, UK; chris.conlon@ndm.ox.ac.uk; 4 TB Chest Clinic, St George’s University Hospitals NHS Foundation Trust, London SW15 5PN, UK; ccosgrov@sgul.ac.uk; 5 Department of Oncology, University of Oxford, Oxford OX3 7DQ, UK; fergus.gleeson@oncology.ox.ac.uk; 6 Royal Free London NHS Foundation Trust, London NW3 2QG, UK; marclipman@nhs.net; 7 UCL Respiratory, University College London, London NW3 2PF, UK; 8 Institute of Immunology and Immunotherapy, University of Birmingham, Birmingham B15 2TT, UK; p.moss@bham.ac.uk; 9 Respiratory Medicine, King’s College Hospital NHS Foundation Trust, London SE5 9PJ, UK; felicity.perrin@nhs.net; 10 Oxford Centre for Respiratory Medicine, Oxford University Hospitals NHS Trust, Oxford OX3 7LE, UK; Henry.Bettinson@ndm.ox.ac.uk

**Keywords:** aerosol vaccine, MVA85A, mycobacteria, latent TB infection

## Abstract

The immunogenicity of the candidate tuberculosis (TB) vaccine MVA85A may be enhanced by aerosol delivery. Intradermal administration was shown to be safe in adults with latent TB infection (LTBI), but data are lacking for aerosol-delivered candidate TB vaccines in this population. We carried out a Phase I trial to evaluate the safety and immunogenicity of MVA85A delivered by aerosol in UK adults with LTBI (NCT02532036). Two volunteers were recruited, and the vaccine was well-tolerated with no safety concerns. Aerosolised vaccination with MVA85A induced mycobacterium- and vector-specific IFN-γ in blood and mycobacterium-specific Th1 cytokines in bronchoalveolar lavage. We identified several important barriers that could hamper recruitment into clinical trials in this patient population. The trial did not show any safety concerns in the aerosol delivery of a candidate viral-vectored TB vaccine to two UK adults with *Mycobacterium tuberculosis* (*M.tb)* infection. It also systemically and mucosally demonstrated inducible immune responses following aerosol vaccination. A further trial in a country with higher incidence of LTBI would confirm these findings.

## 1. Introduction

Tuberculosis (TB) is the leading cause of death from a single infectious agent, with an estimated 10 million new cases and 1.4 million deaths in 2018 [1]. The development of a safe and effective TB vaccine is a key component of pillar 1 of the End TB strategy, which seeks to end the global TB epidemic [2].

The route of a *Mycobacterium tuberculosis* (*M.tb)* infection is by the inhalation of aerosolised infectious droplets, leading to primary infection in the lung and the development of a mucosal immune response [3,4]. In most people, this immune response contains the primary infection and results in latent infection, which carries a 5–10% lifetime risk of developing an active disease [1,5,6]. One-quarter of the global population is estimated to have a latent *M.tb* infection (LTBI).

The only licensed vaccine, Bacille Calmette–Guérin (BCG), offers protection against disseminated TB in childhood, but is less effective against pulmonary TB in many endemic areas [7,8]. A more effective vaccine is urgently needed.

Vaccine delivery by aerosol has potential advantages, including the induction of local immune responses at the site of infection and the practical advantage of needle-free delivery [9,10,11,12,13].

Preclinical animal studies provided proof-of-concept for this aerosol vaccination approach [14,15,16,17]. One candidate vaccine, modified Vaccinia virus Ankara expressing mycobacterial antigen 85A (MVA85A), was shown to be safe and immunogenic as an intradermal or intramuscular booster vaccine in BCG-primed subjects [18,19,20,21,22,23,24]; however, immunogenicity was much weaker in South African infants, and a Phase IIb efficacy trial reported no enhancement of BCG-induced protection [25]. Aerosol delivery may be one way of enhancing immunogenicity. The tolerability and feasibility of administering aerosol MVA85A to humans was evaluated in two Phase I safety trials. In both trials, administering aerosol MVA85A as a boost to a BCG prime was well-tolerated and highly immunogenic [26,27]. Before further evaluation of this route of administration in countries with a high burden of TB, it is important to determine the safety of this approach in subjects with LTBI. We previously demonstrated that intradermal MVA85A was well-tolerated and immunogenic in subjects with LTBI [28,29,30].

Here, we present the results of a Phase I trial to evaluate the safety and immunogenicity of MVA85A vaccination delivered by aerosol in UK adults with LTBI. 

## 2. Materials and Methods

### 2.1. Trial Design

We conducted a Phase I open-label clinical trial in healthy UK adults with latent TB infection (LTBI). All trial documents were approved by the Medicines and Healthcare Regulatory Agency (MHRA, EudraCT 2015-001826-41) and the South Central—Oxford A Research Ethics Committee (reference 15/SC/0370). It was registered with clinicaltrials.gov prior to the start of the study (NCT02532036, 25/08/2015) and conducted according to the principles of the Declaration of Helsinki and good clinical practice. The trial was originally designed with the first 6 volunteers to receive 1 × 10^7^ pfu aerosol-inhaled MVA85A, and the next 24 subjects to be randomised to either 5 × 10^7^ pfu aerosol-inhaled MVA85A with IM saline placebo (Group A) or 5 × 10^7^ pfu IM MVA85A with aerosol-inhaled saline placebo (Group B), with 12 subjects in each group. Due to initial poor recruitment, Group B was taken out of the trial design to ensure that the recruitment numbers would still be sufficient for the primary objective of a safety assessment, and target recruitment for the Starter 1 × 10^7^ pfu group revised down to three volunteers. Group A was subsequently also unable to enrol.

### 2.2. Participants

Participants were healthy adults aged 18–55 with LTBI (defined by positive screening IFN-γ release assay response to ESAT-6 and CFP-10) who had a low risk of reactivation due to a distant contact history, no clinical or radiological features to suggest active TB, and who had not been treated for LTBI. Participants were recruited from TB contact clinics at Oxford University Hospitals, Birmingham Heartlands Hospital, Royal Free Hospital, King’s College Hospital, and St George’s University Hospital (the latter three sites were added after trial commencement due to poor recruitment). Informed consent was obtained for all screened volunteers. 

### 2.3. Vaccines

MVA85A was manufactured under good manufacturing practice conditions by IDT Biologika GmbH, Dessau-Roßlau, Germany. The used dose of MVA85A was 1 × 10^7^ plaque-forming units (pfu). In total, 120 μL of a 1:10 dilution of 8.4 × 10^8^ pfu/mL (starting concentration) using 0.9% sodium chloride was added to the nebuliser, with a further 880 µL of 0.9% sodium chloride added to achieve a final volume of 1 mL. The 1 mL aerosol vaccine was delivered using an Omron MicroAir U22 ultrasonic mesh nebuliser (Omron Healthcare UK, Ltd., Milton Keynes, UK). All vaccinations were performed at the Centre for Vaccinology and Tropical Medicine (CCVTM), University of Oxford.

### 2.4. Clinical Interventions

Volunteers received 1 × 10^7^ pfu MVA85A via aerosol inhalation. Blood was taken at every trial visit (Days 0, 2, 7, 14, 28, and 84) for exploratory immunology; biochemical and haematological parameters were measured at baseline, and Days 7 and 28. Volunteers were followed up to Day 168 post vaccination. Fibreoptic bronchoscopy was performed on all volunteers 7 days after vaccination. Bronchoalveolar lavage (BAL) was obtained from the right-middle lobe using 100 mL of 0.9% sodium chloride. No biopsies were taken.

### 2.5. Objectives

The primary objective was to evaluate the safety of MVA85A vaccination by the aerosol-inhaled route in healthy volunteers latently infected with *Mycobacterium tuberculosis* (*M.tb*).

Safety was assessed by the frequency and severity of adverse events (AEs) during the trial period. Expected respiratory (cough, sore throat, wheeze, dyspnoea, sputum production, haemoptysis, and chest pain) and systemic (fever, feverishness, fatigue, malaise, headache, myalgia, arthralgia, and nausea) AEs were solicited using a diary card for 14 days after vaccination and reviewed at every clinic visit. Volunteers were also asked to report any other AEs experienced over the trial period. Blood biochemical and haematological parameters were measured on Days 7 and 28. Volunteers were trained in the use of a digital thermometer and a handheld spirometer (Micro Spirometer, CareFusion, Chatham, UK). Daily home measurements of temperature, forced expiratory volume in 1 second (FEV_1_), and forced vital capacity (FVC) were recorded for 14 days after vaccination. Vital signs, including pulse oximetry, were taken, and spirometry was performed during clinic visits. A computed-tomography (CT) chest scan was performed prior to enrolment and at day 28. The day 28 scan was compared with the baseline scan by a consultant radiologist at Oxford University Hospitals.

Secondary objectives were to evaluate the systemic and mucosal, cellular and humoral immunogenicity induced by MVA85A. After removal of the intradermal group, this endpoint became descriptive only, with comparison of overall responses between this cohort and data from previous trials.

### 2.6. Ex Vivo Enzyme-Linked ImmunoSpot (ELISpot) 

Peripheral blood mononuclear cells (PBMCs) were isolated from the blood of volunteers on day of screening, day of vaccination (D0), and Days 7, 14, 28, 84 and 168 post-MVA85A vaccination. An IFN-γ ELISpot assay was performed on the freshly isolated PBMC as previously described [27]. Briefly, either 3 × 10^5^ or 1 × 10^5^ PBMCs in 80 µL media were added to triplicate ELISpot wells with 20 µL antigen. Ag85A-specific responses were measured using a single pool of Ag85A peptides (66 15mer peptides, overlapping by 10 amino acids; Peptide Protein research, Bishops Waltham, UK). Antivector responses were measured using separate pools of CD4 (27 14–21mer peptides) and CD8 (36 9mer peptides) epitopes from Vaccinia and MVA (Peptide Protein research, Bishops Waltham, UK; final concentration, 2 μg/mL). Responses to purified protein derivative (PPD) from *M.tb* (Statens Serum Institute, Copenhagen, Denmark; final concentration, 20 μg/mL) and ESAT-6 (17 15mer peptides, overlapping by 10 amino acids) and CFP-10 (18 15mer peptides, overlapping by 10 amino acids; Biomatik; final concentration, 10 µg/mL) were also measured. Staphylococcal enterotoxin B (SEB; Sigma, Kanagawa, Japan; final concentration, 10 μg/mL) was used as a positive control and unstimulated PBMCs as a measure of background IFN-γ production. Results are presented as spot-forming cells (SFCs) per million PBMC, calculated by subtracting the mean of the unstimulated wells from the mean of the antigen-stimulated wells and correcting for the number of PBMCs in the well. 

### 2.7. Enzyme-Linked-Immunosorbent-Assay (ELISA) 

Immunoglobulin G (IgG) levels were measured in serum collected on day of vaccination and Days 7, 14, 28, 84, and 168 post vaccination, as previously described [27]. Briefly, ELISA plates (NUNC ImmunoPlates, Thermo Fisher Scientific, Renfrew, UK) were coated overnight with 2 μg/mL r85A (Lionex, Braunschweig, Germany) in PBS. Samples were diluted 1:10 in 1% casein in PBS (Fisher Scientific) and tested in triplicate. A pool of IgG-positive sera was included in each plate. Wells including all reagents except serum were used to measure the background. Bound serum IgG was detected by goat antihuman γ-chain whole IgG alkaline phosphatase conjugate (Sigma), plates were developed for 30 mins in the dark using a diethanolamine/4-nitrophenylphosphate kit (Sigma) according to the manufacturer’s instructions, and the reaction stopped with 3M NaOH. Absorbance was measured at 405 nm, and mean background-subtracted optical-density (OD) values are presented.

### 2.8. Intracellular Cytokine Staining (ICS) on BAL and Peripheral Blood Mononuclear Cells (PBMCs) 

BAL cells and PBMCs were isolated and stimulated at 1 × 10^6^ cells/mL with Ag85A, ESAT-6/CFP-10 peptides (2 µg/mL each), and PPD (20 µg/mL); unstimulated cells and SEB-stimulated cells were used as negative and positive controls, respectively. Brefeldin A (Sigma) was added to the cells 2 h after stimulation, and cells were incubated overnight at 37 °C and 5% CO_2_.

Harvested BAL cells and PBMCs were stained with the live/dead red viability marker (Thermo Fisher) for the exclusion of dead cells, followed by surface staining with CD4-Pacific Blue (Biolegend, San Diego, CA, USA), CD14, and CD19 on ECD (Beckman Coulter, Brea, CA, USA). Cells were then permeabilised and stained with CD3-AF700 (Ebioscience, San Diego, CA, USA), CD8-APC/H7 (BD), IFN-γ-PECY7 (Ebioscience) and TNF-α-AF-647 (Biolegend), IL-2 PE (Beckman Coulter), and IL-17-AF488 (Biolegend).

Cytokine-producing CD4+ and CD8+ T-cells were gated on CD3+, CD14−, CD19− single T cells. Data were analysed using Flowjo (BD) and are presented as background-subtracted antigen-specific responses.

### 2.9. Statistical Analysis

Clinical AEs were summarised by the frequency and severity of AEs. As only two volunteers were enrolled, immunology results presented below are only descriptive, and no statistical tests were applied. 

## 3. Results

**Figure 1:**
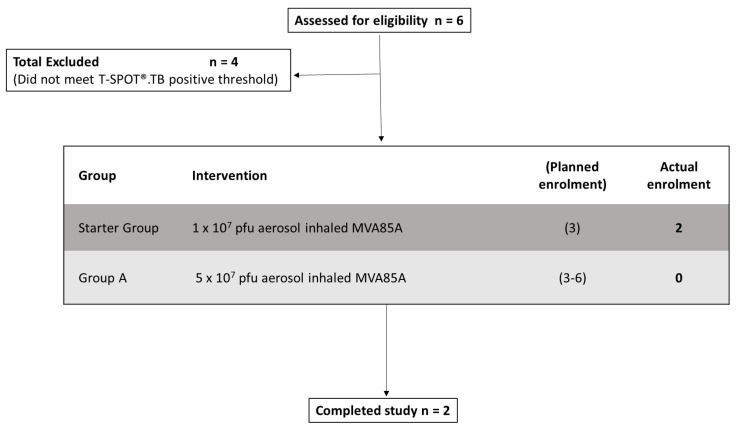
CONSORT flow diagram showing subject recruitment, follow-up, and reasons for exclusion.

Enrolment is summarised in the CONSORT diagram below (Figure 1). Six volunteers were screened for eligibility, and two were recruited into the trial; both completed all scheduled trial visits and procedures. Neither volunteer had a history of BCG vaccination. The trial ran from November 2015 until October 2018 and was terminated due to poor recruitment (see Discussion). 

### 3.1. Safety

There were no serious AEs during the trial; the vaccine was well-tolerated in both volunteers with no symptomatic AEs related to the study product.

There were no clinically significant differences in FEV_1_ or FVC compared to baseline following aerosol vaccination in either volunteer (defined as >15% drop from baseline), except immediately following bronchoscopy. Spirometry returned to normal in both volunteers within 48 h of the bronchoscopy and remained normal at the Day 14 follow-up visit (data not shown). 

For volunteer 002, a new cluster of airway-related nodules in the lateral basal segment of the right lower lobe and the apical segment of the left lower lobe was detected on the Day 28 thoracic CT scan. Changes were reported as consistent with a new minor infection. The volunteer was asymptomatic, with no intercurrent illness in the month from vaccination to CT scan, and there was no evidence of active infection on examination. 

As reactivation of tuberculosis could not be ruled out, the volunteer underwent a second bronchoscopy 6 weeks following vaccination. BAL fluid was collected for mycobacterial culture, *M.tb* PCR, respiratory viral PCR (influenza A and B, and respiratory syncytial virus), cytology, and flow cytometry. The bronchoscopy was macroscopically normal. BAL fluid was negative for all culture and PCR, and cytology was normal. A repeat thoracic CT scan 3 months later showed full resolution of the nodules. These radiological changes were likely due to a transient vaccine response. 

As per protocol, volunteers could start tuberculosis treatment one month after vaccination if advised by their treating clinician as part of their standard LTBI management. Volunteer 301 commenced pyridoxine, rifampicin, and isoniazid 10 weeks (71 days) after vaccination. This treatment plan was in place prior to commencing enrolment and was not related to any trial procedures. Treatment was ongoing at the end of trial.

### 3.2. Ex Vivo IFN-γ Enzyme-Linked Immunospot (ELIspot)

**Figure 2:**
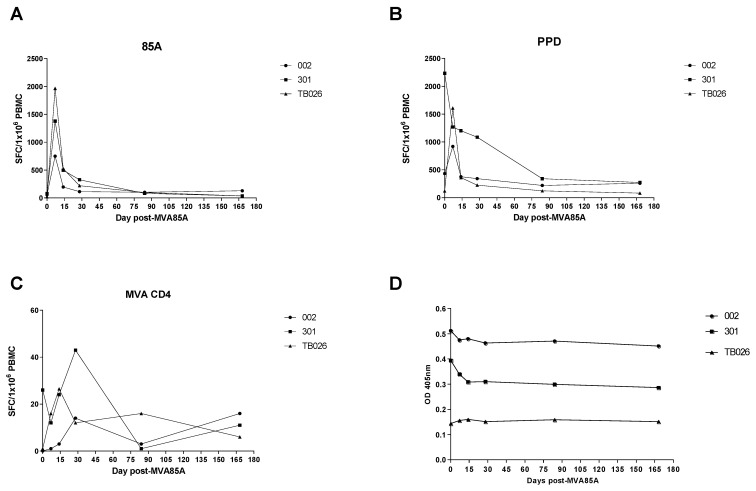
IFNγ enzyme-linked immunospot (ELIspot) responses (**A**–**C**) or anti-r85A IgG levels (**D**). Frequency of antigen-specific IFN-γ ELISpot responses to (**A**) Ag85A, (**B**) purified protein derivative (PPD), and (**C**) antivector MVA CD4; (**D**) IgG levels against recombinant 85A in serum. x axis: time points in days; y axis: (**A**–**C**) spots per 1 × 10^6^ PBMC, (**D**) absorbance at 405 nm. Circles and squares represent individual readings for two latently infected volunteers in this study, and triangles represent median readings for 10 healthy volunteers who received the same dose of aerosol MVA85A from our previous TB026 study [27].

**Figure 3:**
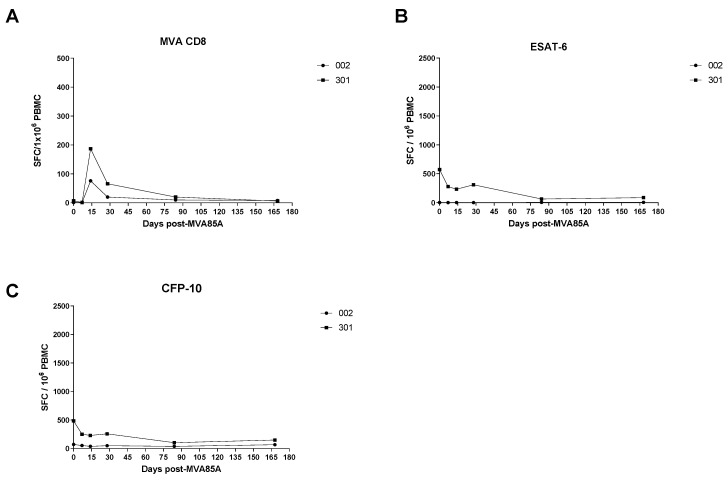
Frequency of antigen-specific IFN-γ ELIspot responses to (**A**) MVA CD8, (**B**) ESAT-6, and (**C**) CFP-10 in two volunteers. *x* axis: timepoints in days; *y* axis: spots per 1 × 10^6^ peripheral blood mononuclear cells.

Both volunteers had high responses in PBMC to Ag85A, which peaked at D7. This is similar to the median responses in healthy volunteers in a previous trial of aerosol MVA85A in healthy UK adults (TB026) [27]; however, both latently infected volunteers had lower peak responses at D7 than the median response of the healthy volunteers at this time point was (Figure 2A). For Volunteer 002, responses to PPD followed the same trend as that in Ag85A, but Volunteer 301 had a high baseline response to PPD, which declined after vaccination (Figure 2B). Antivector responses to MVA CD4+ and MVA CD8+ T-cell epitopes were similar to those seen in healthy volunteers from TB026 (Figure 2C and Figure 3). Responses to ESAT-6 and CFP-10 peptides were variable. Volunteer 002 only responded to CFP-10 peptides, and responses did not change through the trial. Volunteer 301 responded to both sets of peptides and appeared to have a decline in responses after vaccination, similar to the trend seen in their PPD responses (Figure 3).

### 3.3. Enzyme-Linked Immunosorbent Assay (ELISA)

Serum IgG levels against r85A did not increase from day of vaccination at any of the time points measured in either of the volunteers (Figure 2D). This is consistent with our previous trial of aerosol MVA85A (TB026) at the same dose in 10 healthy volunteers [27]. However, both latently infected volunteers had considerably higher OD values throughout the time course than the median OD values in healthy volunteers.

### 3.4. BAL and PBMC Intracellular Cytokine Responses

**Figure 4:**
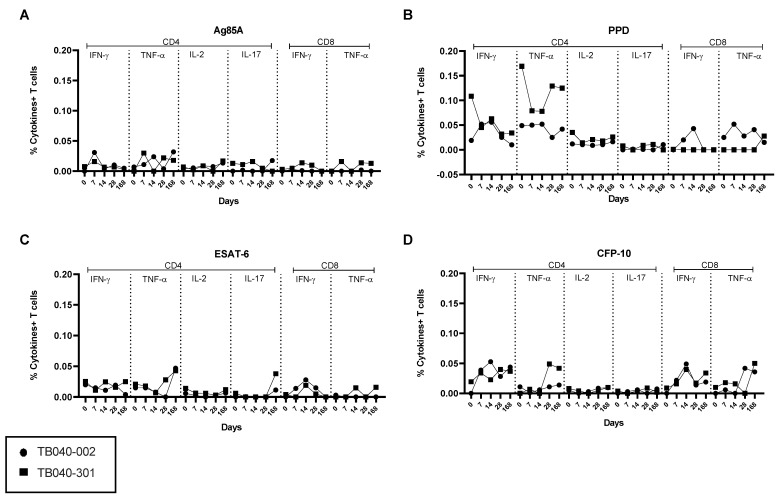
Peripheral blood mononuclear cell (PBMC) intracellular cytokine staining (ICS). PBMC ICS antigen-specific responses in two latent *Mycobacterium tuberculosis* (*M.tb)*-infected UK adults vaccinated with 1 × 10^7^ pfu aerosol-inhaled MVA85A. Percentages of CD4+ T cells producing IFN-γ, TNF-α, IL-2, IL-17, and CD8+ T cells producing IFN-γ and TNF-α in response to (**A**) Ag85A, (**B**) PPD, (**C**) ESAT-6, and (**D**) CFP-10. Individual values shown for each volunteer.

**Figure 5:**
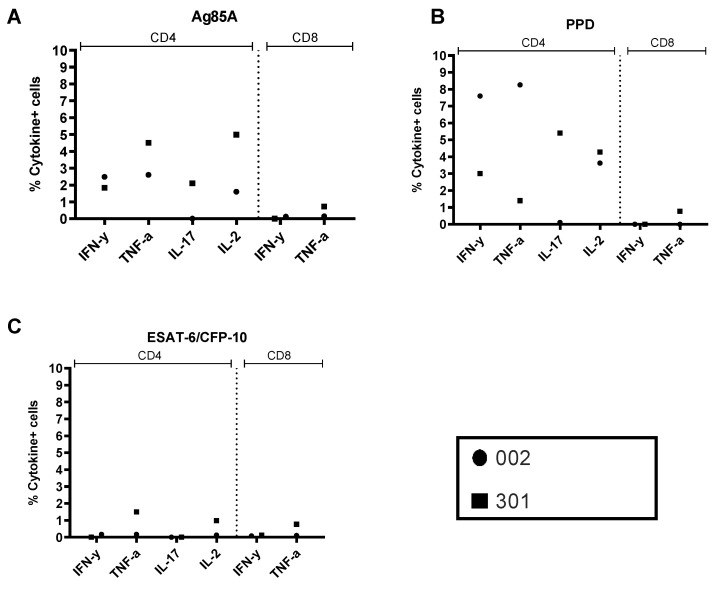
Bronchoalveolar lavage (BAL) intracellular cytokine staining (ICS) 7 days after aerosol MVA85A vaccination. BAL ICS antigen-specific responses in two latent *Mycobacterium tuberculosis* (*M.tb)*- infected UK adults vaccinated with 1 × 10^7^ pfu aerosol-inhaled MVA85A. Percentages of CD4+ T cells producing IFN-γ, TNF-α, IL-2, IL-17, and CD8+ T cells producing IFN-γ and TNF-α in response to (**A**) Ag85A, (**B**) PPD, and (**C**) ESAT-6/CFP-10. Individual values shown for each volunteer.

Antigen-specific PBMC intracellular cytokine responses were detected at Days 0, 7, 14, 28, and 168. IFN-γ and TNF-α were detected in both CD4+ and CD8+ T cells, while IL-2 and IL-17 were only detectable in CD4+ T cells (Figure 4). In BAL, 7 days following vaccination, the frequencies of PPD-specific cytokine+ CD4+ T cells were higher than the Ag85A-specific responses (Figure 5). CD4+ IFN-γ, TNF-α, and IL-2 were detected in both volunteers, and IL-17 was detected in one volunteer. Low levels of PPD and Ag85A-specific CD8+ T-cell cytokines were detected in one volunteer, as were mucosal ESAT-6/CFP-10-specific CD4+ TNF-α, IL-2, and CD8+ TNF-α. For both volunteers, the PPD-specific CD4+ IFN-γ + response was higher in BAL compared to PBMC at the same time point (3.01% or 7.6% of total BAL CD4+ T cells compared to 0.045% or 0.052% of total PBMC CD4+ T cells; Figure 4 and Figure 5).

## 4. Discussion

The aerosol inhalation of low-dose (1 × 10^7^ pfu) MVA85A in two volunteers with LTBI was well-tolerated with no clinically significant adverse events (AEs). The only AEs likely related to the vaccine were transient asymptomatic microbial negative (PCR and culture) radiological changes to the lung on the Day 28 CT scan in one volunteer.

Delivering aerosol MVA85A to adults with LTBI induced mycobacterium- and vector-specific IFN-γ, and mycobacterium-specific mucosal Th1 cytokines in the BAL and peripheral blood; however, no humoral response to Ag85A was detectable in either compartment following this route of immunisation. 

For Volunteer 301, anti-TB treatment was commenced at Day 71 post vaccination. While the numbers are small, making it difficult to draw conclusions from the immune data between the two volunteers, treatment at this time point did not make any obvious difference to the trends seen in either local or systemic immune responses for Volunteer 301.

The trial was open for recruitment for 3 years in up to six sites. However, due to significant challenges with recruitment at all UK sites, this study was closed before the completion of any group. The lower dose of 1 × 10^7^ pfu in this study was planned as a starting dose for safety reasons, with the target dose of 5 × 10^7^ pfu designed to provide further information on safety and immunogenicity. Due to recruitment shortages, we were unable to test our planned target dose of 5 × 10^7^ pfu MVA85A. Doses of between 5 × 10^7^ and 1 × 10^8^ pfu have been established as an intradermal dose in healthy and LTBI adults [28,29,30]. While 1 × 10^8^ pfu aerosol MVA85A has been given to *M.tb*-naive UK adults, doses between 1 × 10^7^ and 5 × 10^7^ pfu are preferred due to the high mucosal cellular immune responses induced by this route [26,27]. Interestingly, in *M.tb*-naive UK adults, there was no substantial difference in the strength of the immune response (ELIspot or ICS) between the 1 × 10^7^ and 5 × 10^7^ pfu aerosol dose at 1 week (while acknowledging that comparison is limited, as this was across two separate trials) [26,27]; hence, the lower dose used here is still informative. Regardless, the low recruitment numbers mean that further early-phase safety and immunogenicity testing is necessary in a TB-endemic area before the wide-scale testing of this immunisation route can be undertaken.

Most potential volunteers were excluded prior to screening due to medical reasons, particularly pregnancy, lactation, smoking, and respiratory disease. Another significant barrier was language, with most potential volunteers having little to no English proficiency. We recruited one Spanish speaker and provided interpreting services and document translation, but if volunteers spoke no English at all, they were excluded due to the rigorous study requirements around safety and data quality. A further major barrier was the inability to take time away from work commitments. Despite amendments to study visits to reduce the time burden of trial participation, recruitment did not improve. 

## 5. Conclusions

In this trial, we demonstrated that, in two latently *M.tb*-infected volunteers, the delivery of aerosolised MVA85A vaccine was well-tolerated with no safety concerns. The aerosol delivery of this candidate viral-vectored TB vaccine systemically induced mycobacterium-specific Th1 cytokines in the local mucosa, and mycobacterium- and vector-specific IFN-γ. The trial highlights the difficulty in carrying out an interventional Phase I clinical trial in healthy latently *M.tb*-infected subjects in the UK. A further trial, conducted in a country with a high burden of TB, would support and extend the findings reported here. 

## Data Availability

The data presented in this study are available on request from the corresponding author. The data are not publicly available due to privacy restrictions.
